# Next-generation T cell–activating vaccination increases influenza virus mutation prevalence

**DOI:** 10.1126/sciadv.abl5209

**Published:** 2022-04-06

**Authors:** Maireid B. Bull, Haogao Gu, Fionn N. L. Ma, Liyanage P. Perera, Leo L. M. Poon, Sophie A. Valkenburg

**Affiliations:** 1 HKU-Pasteur Research Pole, School of Public Health, Li Ka Shing Faculty of Medicine, The University of Hong Kong, Hong Kong SAR, China.; 2 Division of Public Health Laboratory Sciences, School of Public Health, Li Ka Shing Faculty of Medicine, The University of Hong Kong, Hong Kong SAR, China.; 3 Metabolism Branch, Center for Cancer Research, National Cancer Institute, National Institutes of Health, Bethesda, MD 20892-1374, USA.; 4 Department of Microbiology and Immunology, at The Peter Doherty Institute for Infection and Immunity, The University of Melbourne, Melbourne, Victoria, Australia.

## Abstract

To determine the potential for viral adaptation to T cell responses, we probed the full influenza virus genome by next-generation sequencing directly ex vivo from infected mice, in the context of an experimental T cell–based vaccine, an H5N1-based viral vectored vaccinia vaccine Wyeth/IL-15/5Flu, versus the current standard-of-care, seasonal inactivated influenza vaccine (IIV) and unvaccinated conditions. Wyeth/IL-15/5Flu vaccination was coincident with increased mutation incidence and frequency across the influenza genome; however, mutations were not enriched within T cell epitope regions, but high allele frequency mutations within conserved hemagglutinin stem regions and PB2 mammalian adaptive mutations arose. Depletion of CD4^+^ and CD8^+^ T cell subsets led to reduced frequency of mutants in vaccinated mice; therefore, vaccine-mediated T cell responses were important drivers of virus diversification. Our findings suggest that Wyeth/IL-15/5Flu does not generate T cell escape mutants but increases stochastic events for virus adaptation by stringent bottlenecks.

## Teaser

Next-generation T cell–activating influenza vaccine stochastic immune pressure is greater than inactivated vaccines.

## INTRODUCTION

The widely used current seasonal inactivated influenza vaccine (IIV) primarily mediates protection by eliciting strain-specific antibodies directed toward the hemagglutinin (HA) head region on the viral surface, which can effectively block infection when the vaccine and circulating strain are antigenically well matched. Yet, current IIVs provide little to no protection against mismatched viruses or pandemic strains; therefore, next-generation influenza vaccines that can provide broad durable and cross-reactive immunity against diverse influenza viruses are urgently needed (*1*). Inactivated vaccines are unable to fully engage major histocompatibility complex (MHC) class I and II processing machinery and, as such, generate suboptimal T cell immunity. While licensed live attenuated influenza vaccine (LAIV) can increase influenza-specific T cell responses in children up to 12 years of age, it has failed to have a boosting effect in adults (*2*), increase protection compared with IIV (*3*), or provide heterosubtypic protection (*4*). A universal next-generation vaccinia vectored vaccine candidate, MVA-NP+M1, currently in phase 2b trials (*5*), has been shown to boost T cell responses, including in adults more than 65 years of age, and led to reduced infection and viral shedding (*6*). As cross-reactive T cells toward H5N1, H7N9, and pandemic H1N1 viruses can be found in the majority of healthy unexposed individuals (*7*, *8*), these responses could be boosted or established by next-generation T cell–activating vaccines potential for improved breadth of protection against diverse influenza viruses.

Influenza A virus (IAV) is a highly mutable virus, as evidenced by continual antigenic drift, and antiviral escape that has rendered some drugs ineffective (*9*). Influenza virus diversification and adaptation have also been recently observed in an asthmatic mouse model (*10*) due to effects on the type I interferon response and for HA stem antibodies in human challenge studies (*11*). While T cell–targeted vaccines seem to be an optimal strategy for universal influenza vaccination, there is a potential for immune escape by viral adaptations. This is due to the fundamental recognition of viral peptides in MHC on infected cells by T cells; therefore, T cell–activating vaccines will likely not provide total sterilizing immunity, and a residual pool of viruses may be under selection pressure. T cell escape mutants generated in response to immune pressure have been observed in other viral infections such as hepatitis C virus and HIV (*12*, *13*) and influenza viruses (*14*–*16*).

Evidence from human influenza virus sequences shows that known T cell epitopes are under positive selective pressure but are typically functionally constrained (*17*). An anchor mutation reducing MHC I binding was identified in the HLA-B*27–restricted NP_383_ epitope in an immunocompromised infant with longitudinal infection and in consensus influenza virus sequences in the mid-1990s leading to a loss of epitope immunogenicity (*18*, *19*). Furthermore, in a mouse infection model, a virus escape mutation in an anchor position of an immunodominant CD8^+^ T cell response (D^b^NP_366–374_-N5H) arose in the majority of mice, early during acute infection, and increased in frequency with time after infection, with limited de novo T cell responses recalled or established to the mutant epitope. In the same model, other CD8^+^ T cell epitopes were subjected to less pressure (D^b^PA_224–233_) or remained conserved in other mouse strains (K^d^NP_147–155_) (*14*). Similarly, a public T cell receptor (TCR) that recognizes the extremely conserved A2-M1_58–66_ CD8^+^ T cell epitope can also recognize the majority of natural variants, which may prevent immune escape (*20*), while unique TCR signatures for B7/B35-NP_418–426_ peptide variants may propagate peptide variation (*20*).

Influenza-specific CD4^+^ T cells are an important cornerstone to protection by next-generation universal influenza vaccines (*21*). The peptide specificity of CD4^+^ T cells in recognition of IAV is less understood because of their broad range of capabilities and roles within the adaptive immune response (*22*), from direct cytotoxicity (*23*–*25*) to indirect helper functions (*26*), and flexible MHC anchor positions; therefore, pinpointing peptide specificities by prediction tools has been more difficult. Therefore, experimental evidence of the role of CD4^+^ T cell–mediated immune pressure for influenza viruses is limited.

Our knowledge is currently limited to inferences of immune escape for sequence databanks of human influenza isolates (*15*, *27*) and limited animal studies (*10*, *14*), which may underestimate the frequency of influenza T cell escape mutations. Less is known about within-host diversity during influenza infection (*28*) and the role of T cell–mediated immune pressure, especially in the context of universal vaccines that can be nonsterilizing compared to the standard of care, IIVs. Therefore, we used next-generation sequencing (NGS) techniques to identify low-frequency variants at an individual level in the context of current inactivated and our well-characterized next-generation T cell–activating vaccine, Wyeth/IL-15/5Flu (*29*), during influenza virus infection.

Wyeth/IL-15/5Flu, containing H5N1-derived IAV proteins, has been previously shown to generate high antibody levels at 28 days after first dose, which protected against lethal challenge H5N1 challenge (*29*). The Wyeth/IL-15/5Flu vaccine is composed of five proteins from H5N1 viruses: haemagglutinin (HA), neuraminidase (NA), and nucleoprotein (NP) derived from A/Vietnam/1203/2004, alongside M1 and M2 derived from A/CK/Indonesia/PA/2003 virus (*29*). Subsequent studies identified that heterosubtypic protection against H1N1, H3N2, and H7N7 was mediated by T cell protection (*21*, *30*). While the Wyeth/IL-15/5Flu does stimulate both the B and T cell arms of the immune system, and as such, selection pressure induced by B cell activation cannot be ruled out, this study will characterize T cell–mediated pressure as they have been indicated as the main driver of protection in this context.

## RESULTS

### Wyeth/IL-15/5Flu generates comparable humoral protection to S-QIIV and superior T cell responses

**Fig. 1.**
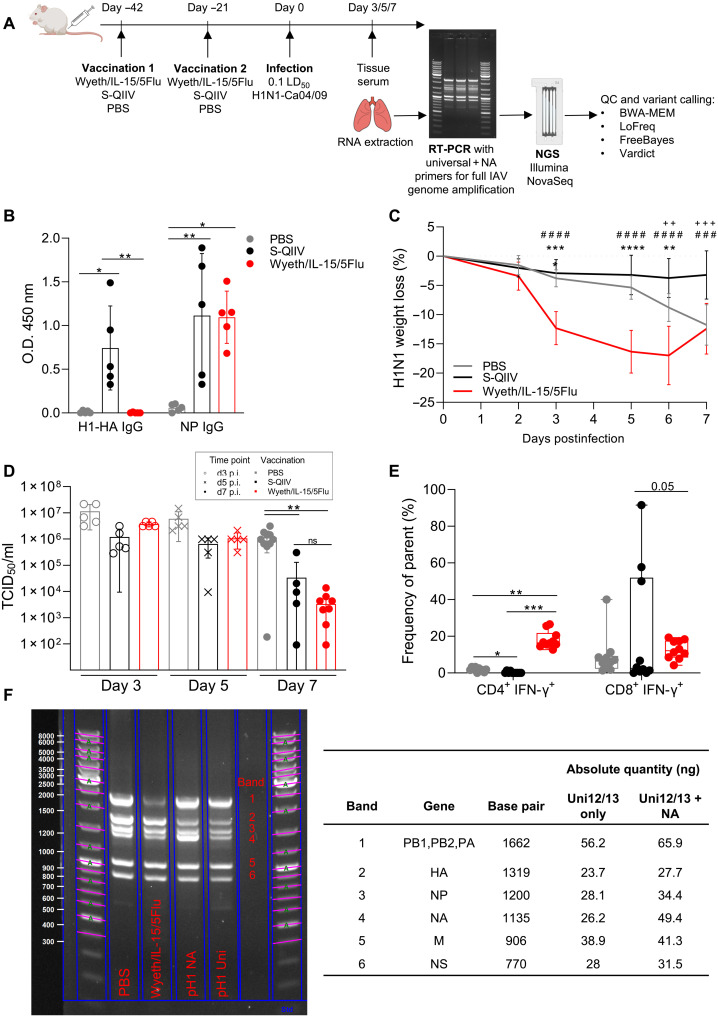
Full IAV genome sequencing from a mouse model of vaccination and infection. (**A**) Mice were vaccinated with PBS, Wyeth/IL-15/5Flu, or S-QIIV twice 21 days apart and challenged with H1N1. Infected lungs were harvested at day 3, 5, or 7 postinfection, and IAV RNA was extracted, amplified by RT-PCR, sequenced by NovaSeq, and analyzed. QC, quality control. (**B**) Total IgG responses in postvaccination BALB/c serum to H1N1 A/California/04/2009 NP and H1-HA protein (*n* = 5, ±SD). O.D., optical density. (**C**) Weight loss during H1N1 infection was monitored (*n* = 10, ±SD), and significance was determined by two-way analysis of variance (ANOVA) (* denotes comparison between S-QIIV versus Wyeth/IL-15/5Flu, # denotes PBS versus Wyeth/IL-15/5Flu, and + denotes PBS versus S-QIIV). (**D**) Lung viral loads by TCID_50_ assay (d3 and d5, *n* = 5; d7, *n* = 10 ± SD). (**E**) H1N1-specific IFN-γ^+^ CD4^+^ CD8^+^ T cell populations from BAL fluid at d7 p.i. (*n* = 10, individual data points shown on box and whiskers, showing mean and upper and lower quartiles with minimum-maximum range). (**F**) A representative full-genome RT-PCR using Uni12/13+NA primer set (PBS, Wyeth/IL-15/5Flu, and pH1 NA) and Uni12/13 primers alone (pH1 Uni) from RNA extracted directly ex vivo from infected lungs or pH1 (positive control H1N1 virus) shows band sizes corresponding to IAV genes quantified by Image Lab (nanogram) in reference to the standard ladder. *P < 0.05, **P < 0.01, ***P < 0.005, and ****P < 0.0001.

Mice were vaccinated twice 21 days apart with phosphate-buffered saline (PBS), seasonal quadrivalent inactivated influenza vaccine (S-QIIV), or Wyeth/IL-15/5Flu and infected with a nonlethal dose of IAV (H1N1). IAV RNA was extracted from supernatants of lung homogenates of infected mice and subjected to whole virus genome reverse transcription polymerase chain reaction (RT-PCR) for Illumina NovaSeq NGS and further downstream analysis (Fig. 1A).

Comparison of postvaccination antibody immunoglobulin G (IgG) responses to H1-HA and NP (Fig. 1B) showed a similar level of NP-specific humoral immunity generated, while only S-QIIV generated H1-HA responses because of matched vaccine content, whereas Wyeth/IL-15/5Flu contains H5-derived HA. After H1N1 challenge, Wyeth/IL-15/5Flu–vaccinated mice had early weight loss compared with PBS- and S-QIIV–vaccinated groups (Fig. 1C). Lung viral loads were comparable between groups at days 3 and 5 postinfection (p.i.) (Fig. 1D). Day 7 viral titers showed a significant decrease in virus load in both S-QIIV and Wyeth/IL-15/5Flu vaccination groups compared with unvaccinated controls (*P* = 0.004 versus S-QIIV, *P* = 0.007 versus Wyeth/IL-15/5Flu) and were not significantly different between S-QIIV and Wyeth/IL-15/5Flu. Comparison of recall of T cell populations to the site of infection (fig. S1) between vaccination groups showed a significant increase in H1N1-specific CD4^+^ T cells in Wyeth/IL-15/5Flu–vaccinated mice versus the PBS- or S-QIIV group (Fig. 1E), while recall of H1N1-specific CD8^+^ T cell populations was borderline significantly higher (*P* = 0.058) in Wyeth/IL-15/5Flu–vaccinated groups versus S-QIIV. Quality of T cell responses was also elevated in Wyeth/IL-15/5Flu–vaccinated mice, with a significant increase in frequency of CD4^+^ and CD8^+^ double and triple producers of interferon-γ (IFN-γ), interleukin-2 (IL-2), and tumor necrosis factor–α (fig. S2).

The aim of this study was to determine the rate of influenza viral genome variation following infection under vaccinated conditions; therefore, we performed whole IAV genome NGS directly ex vivo from infected mouse lungs. Following successful amplification of all IAV genes with universal and spiked-in NA-specific primers, the subsequent RT-PCR products were quantified (nanograms) using Bio-Rad Image Lab Software to ensure suitability for downstream NGS (Fig. 1F). A representative RT-PCR gel is shown from day 7 p.i. PBS and Wyeth/IL-15/5Flu lungs with Uni12/13+NA primers and positive control (pH1) with Uni12/13+NA primers (pH1 NA) or universal primers only (Uni12/13). Spiking in of NA-specific primers improves NA band quality and later sequencing read coverage and was therefore the NA primer used in combination with Uni12/13 primers for full genome coverage (fig. S3).

### T cell–activating vaccine induces a higher incidence and degree of mutation on the IAV genome

NGS samples were subjected to an analysis pipeline for low-frequency variant calling, single-nucleotide polymorphisms (SNPs) <0.01 allele frequency (AF; equivalent <1%) were excluded, and those that achieved sufficient read coverage up to day 7 of infection were included for analysis (Table 1). Sequences were disqualified from further analysis that had <100 reads at any nucleotide position or <0.8 Q33 Phred score; for example, at the day 7 time point, one of nine mice in PBS, one of five mice S-QIIV, and two of eight mice for Wyeth/IL-15/5Flu groups were excluded (fig. S4).

**Table 1.** Average read depth per gene after Illumina NovaSeq sequencing. Average read depth and Q33 Phred score listed across all eight IAV gene segments in PBS-, S-QIIV–, and Wyeth/IL-15/5Flu–vaccinated BALB/c mice at the day 3, 5, and 7 time points. Samples with below average 100 read depth in any gene or <0.8 Q33 Phred score were removed from further analysis. Individual mouse data coverage is shown in fig. S1.

			**Reads/gene**											
**Group**	**Sample**	**Total** **reads**	**HA**	**NA**	**NP**	**PA**	**PB1**	**PB2**	**NS**	**M**	**Q33** **forward** **read**	**Q33** **reverse** **read**	**Total** **samples**	**Total** **excluded** **from (*n*** **of *Z*)**
PBS	Day 3	1299984	275221	54302	129188	17404	115661	5302	346956	355950	0.95	0.91	5	0 of 5
	Day 5	1384659	327416	34054	152365	26725	160586	6485	296320	380708	0.93	0.91	5	0 of 5
	Day 7	1276823	256557	52795	129599	25269	123572	6248	346107	336676	0.95	0.89	5	1 of 5
S-QIIV	Day 3	1336142	332069	52081	129483	9977	99752	6323	331025	375432	0.95	0.92	5	0 of 5
	Day 5	1305060	165459	51008	100765	14054	37590	5601	525265	405318	0.96	0.92	5	0 of 5
	Day 7	449783	56171	27173	34324	6020	15002	3019	196960	111114	0.92	0.91	5	0 of 5
Wyeth/ IL-15/ 5Flu	Day 3	1389717	138224	98902	108375	11177	22754	3809	580430	426046	0.89	0.94	9	1 of 9
	Day 5	1328671	139208	42190	70365	10879	25379	3542	640783	396325	0.95	0.89	6	2 of 6
	Day 7	124991	4859	5269	4086	590	2481	227	71363	36116	0.93	0.88	8	2 of 8

**Fig. 2.**
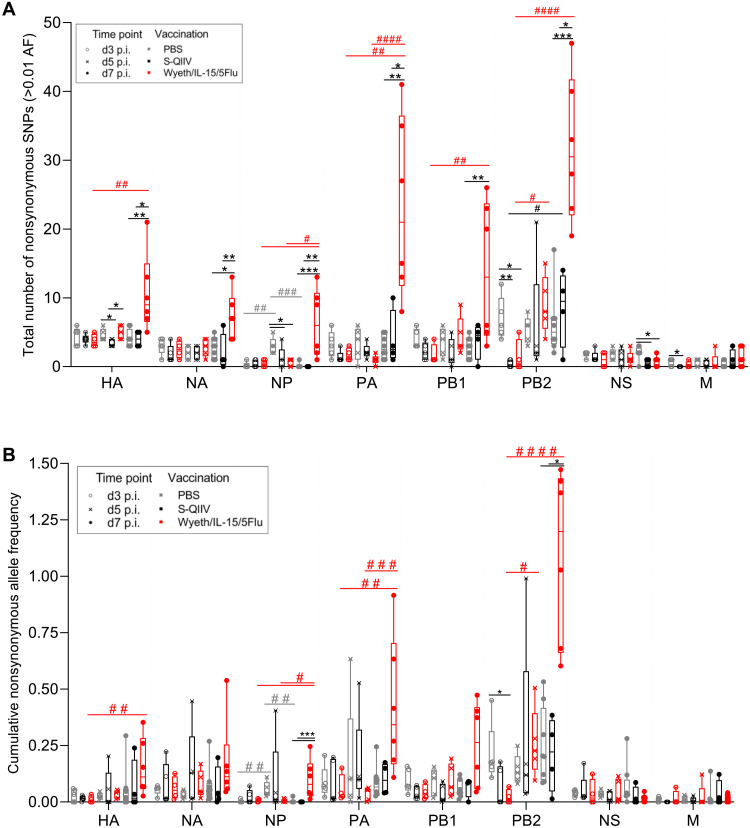
Wyeth/IL-15/5Flu increases the nonsynonymous SNP incidence and cumulative AF on the IAV genome. (**A**) Total number of nonsynonymous SNPs and (**B**) cumulative AF arising in IAV gene sequences isolated from H1N1 days 3, 5, and 7 p.i. BALB/c mice vaccinated with PBS, S-QIIV, or Wyeth/IL-15/5Flu across all eight IAV genes (≥0.01 AF). Individual data points shown on box and whiskers plot, showing mean and upper and lower quartiles with minimum-maximum range. Statistical significance was determined by a Kruskal-Wallis test. False discovery rate (0.1) was determined by the original Benjamini and Hochberg method. * denotes significance between different vaccination groups at same time point, and # denotes significance between different time points within the same vaccination group. */#*P* ≤ 0.05, **/##*P* ≤ 0.01, ***/###*P* ≤ 0.001, and ####*P* ≤ 0.0001.

Comparison of the total number of nonsynonymous SNPs arising at ≥0.01 AF across eight IAV genes between vaccination groups showed that Wyeth/IL-15/5Flu vaccination increased mutational incidence across multiple IAV genes (Fig. 2A). The most drastic increase was observed on day 7, where Wyeth/IL-15/5Flu–vaccinated mice had significantly higher SNP incidence (i.e., count of any SNP regardless of frequency, ≥0.01 AF) than both PBS- and S-QIIV–vaccinated groups in five of eight genes: HA, NA, NP, polymerase acidic (PA), and polymerase basic 2 (PB2). There was also an increase in mutations over time, from day 3 to day 7 of infection, in Wyeth/IL-15/5Flu–vaccinated groups within HA, NP, PA, and PB2 genes, whereas sequences from S-QIIV vaccination groups remained stable with time when compared with PBS.

Furthermore, when the cumulative AF (i.e., sum of AF within an individual mouse across a gene at ≥0.01 AF) was investigated, it was observed that mutations arising in Wyeth/IL-15/5Flu were also occurring at a higher frequency compared with PBS- and S-QIIV–vaccinated mice (Fig. 2B). This significant increase was seen at day 7 in two of eight genes: NP and PB2. Whereas S-QIIV vaccination did not lead to significantly higher cumulative AF versus PBS at any time point within any gene, Wyeth/IL-15/5Flu–vaccinated mice accumulated mutations over time when comparing between day 3 and day 7 samples, significantly increasing their mutational load in four of eight genes: HA, NP, PA, and PB2.

### T cell depletion abates mutational increases driven by a T cell–activating vaccine

**Fig. 3.**
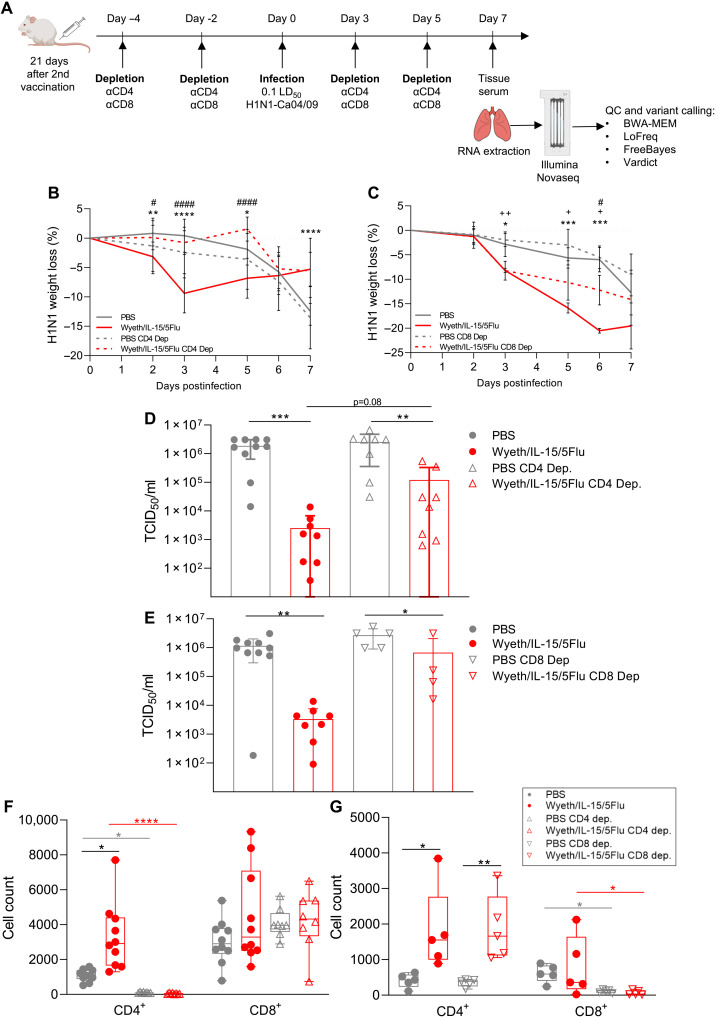
T cell depletion of BALB/c mice and subsequent challenge with H1N1. (**A**) T cell depletion schedule of PBS- or Wyeth/IL-15–vaccinated mice. Infected lungs were harvested at day 7 postinfection, and IAV RNA was extracted, amplified by RT-PCR, sequenced by NovaSeq, and analyzed. Weight loss during H1N1 infection was monitored in (**B**) PBS and Wyeth/IL-15/5Flu nondepleted (*n* = 10 ± SD) and CD4^+^ T cell depleted (*n* = 8 ± SD). (**C**) PBS and Wyeth/IL-15/5Flu nondepleted (*n* = 10 ± SD) and CD8^+^ T cell depleted (*n* = 5 ± SD). Significance was determined by two-way ANOVA (* denotes comparison between nondepleted PBS versus Wyeth/IL-15/5Flu, # denotes nondepleted Wyeth/IL-15/5Flu versus T cell–depleted Wyeth/IL-15/5Flu, and + denotes T cell–depleted PBS versus Wyeth/IL-15/5Flu). Lung viral loads (±SD) by TCID_50_ assay comparison of nondepleted PBS and Wyeth/IL-15/5Flu versus (**D**) CD4^+^ T cell–depleted and (**E**) CD8^+^ T cell–depleted PBS and Wyeth/IL-15/5Flu–vaccinated groups. H1N1-specific IFN-γ^+^ CD4^+^ CD8^+^ T cell populations from BAL fluid at day 7 p.i. in (**F**) CD4^+^ and (**G**) CD8^+^ T cell–depleted conditions. Individual data points shown on box and whiskers plot, showing mean and upper and lower quartiles with minimum-maximum range. Statistical significance was determined by a Kruskal-Wallis test. False discovery rate (0.1) was determined by the original Benjamini and Hochberg method. *P < 0.05, **P < 0.01,***P < 0.005, and ****P < 0.0001.

T cell depletion of vaccinated mice at infection was performed to determine the impact of enhanced T cell responses by Wyeth/IL-15/5Flu vaccination on the increased mutational frequency observed after IAV challenge (Fig. 3A). CD4^+^ T cell depletion led to comparable overall weight loss in Wyeth/IL-15/5Flu–vaccinated mice during H1N1 challenge by day 7 but had reduced weight loss by comparison at early infection time points, while unvaccinated mice had greater weight loss by day 7 (Fig. 3B). CD8^+^ T cell–depleted mice after H1N1 challenge had less weight loss compared with nondepleted counterparts (Fig. 3C). Lung viral loads of CD4^+^ T cell–depleted (Fig. 3D) and CD8^+^ T cell–depleted (Fig. 3E) vaccination groups showed no significant difference between Wyeth/IL-15/5Flu–vaccinated groups at day 7 p.i. However, Wyeth/IL-15/5Flu vaccination still significantly reduced viral titers even under CD4^+^ or CD8^+^ T cell–depleted conditions compared with PBS-vaccinated T cell–depleted mice. Recall of T cell responses to the lung, by fluorescence-activated cell sorting analysis of bronchoalveolar lavage (BAL) samples, showed that CD4^+^ (Fig. 3F) and CD8^+^ (Fig. 3G) T cell responses were ablated by depletion (74.5 to 99.8% and 99.1 to 99.2%, respectively). T cell responses were otherwise recalled and increased by Wyeth/IL-15/5Flu vaccination.

**Fig. 4.**
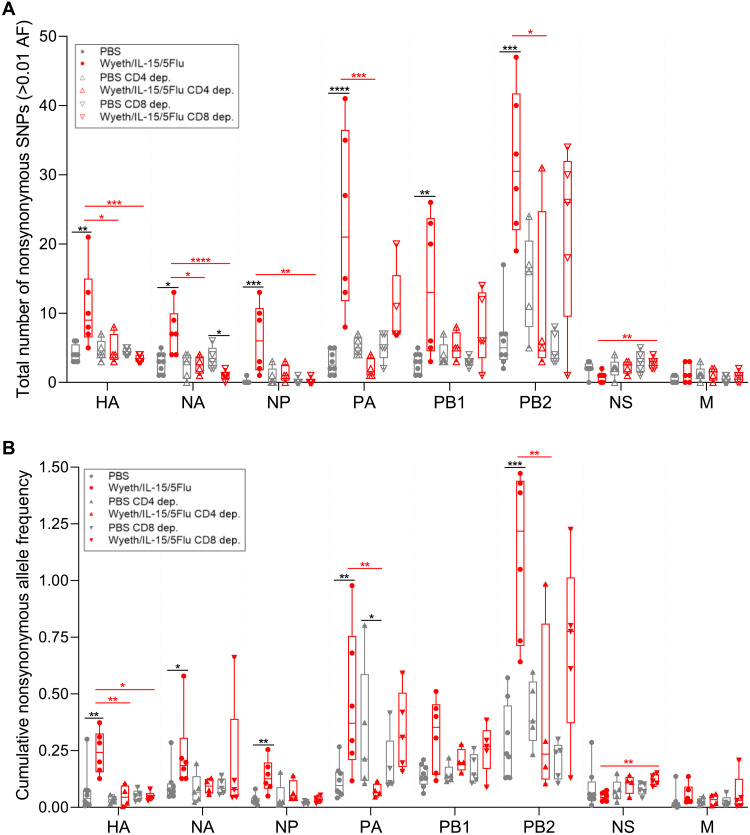
CD4 and CD8 depletion leads to reduced mutations in Wyeth/IL-15/5Flu–vaccinated mice at day 7 after H1N1 challenge. (**A**) Total number of nonsynonymous SNPs and (**B**) cumulative AF arising in IAV gene sequences isolated from H1N1 day 7 p.i. nondepleted (same sequence data from Fig. 2), CD4^+^ and CD8^+^ T cell–depleted BALB/c mice vaccinated with PBS or Wyeth/IL-15/5Flu, across all eight IAV genes (≥0.01 AF). Individual data points shown on box and whisker plots, showing mean and upper and lower quartiles with minimum-maximum range. Statistical significance was determined by a Kruskal-Wallis test. False discovery rate (0.1) was determined by the original Benjamini and Hochberg method. Black annotation denotes significance between different vaccination groups at the same time point; red annotation denotes significance between different depletion status within the same vaccination group. *P ≤ 0.05, **P ≤ 0.01, ***P ≤ 0.001, and ****P ≤ 0.0001. PCR and sequencing were performed in parallel for the control nondepleted group of day 7 from Fig. 2; therefore, the same nondepleted PBS and Wyeth/IL-15/5Flu control samples were used in Fig. 2 and in this figure.

Analysis of SNP incidence in T cell–depleted groups showed a significant reduction of mutations in Wyeth/IL-15/5Flu–depleted groups compared with nondepleted vaccinated mice (Fig. 4A). In Wyeth/IL-15/5Flu–vaccinated mice, CD4^+^ depletion led to a reduced SNP incidence in four of eight genes: HA, NA, PA, and PB2. Furthermore, in Wyeth/IL-15/5Flu–vaccinated mice, CD8^+^ depletion led to reduced mutations in HA, NA, and NP genes and significantly increased mutations in non-structure (NS) genes. Furthermore, CD8^+^ depletion also significantly reduced NA SNPs in Wyeth/IL-15/5Flu mice compared with PBS. Overall, nondepleted Wyeth/IL-15/5Flu–vaccinated mice induced significantly higher level of SNPs compared with nondepleted PBS vaccination groups across the majority of IAV genes (Fig. 2A), whereas in T cell–depleted groups, Wyeth/IL-15/5Flu SNP incidence was comparable to PBS.

In Wyeth/IL-15/5Flu–vaccinated mice, both CD4^+^ and CD8^+^ T cell depletion significantly reduced the cumulative AF of the HA gene (Fig. 4B). In addition, CD4^+^ T cell depletion significantly reduced cumulative AF of both PA and PB2 genes, whereas CD8^+^ T cell depletion of Wyeth/IL-15/5Flu–vaccinated mice did not lead to significant reductions in degree of mutation outside of the HA gene and significantly increased cumulative mutations within NS.

### Mutations arising in T cell–activated vaccinated conditions have the capacity to be beneficial to IAV replication and evasion of host immune responses

**Fig. 5.**
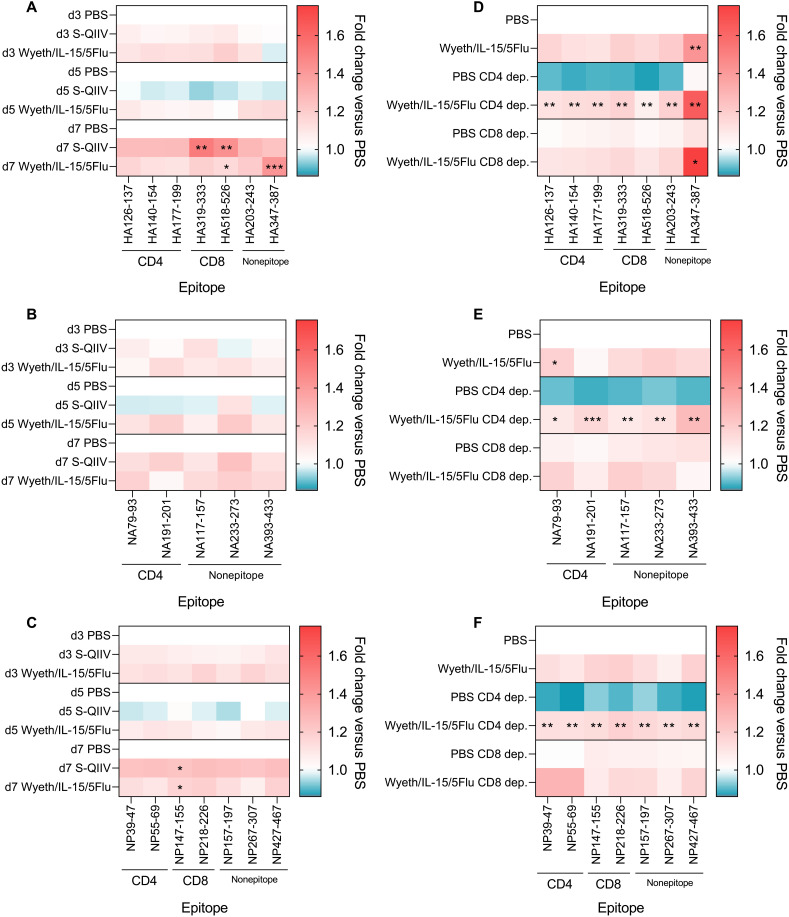
Wyeth/IL-15/5Flu–induced immune pressure does not induce widespread mutations within T cell epitope regions. Fold change of nucleotide base diversity versus appropriate control PBS groups of known BALB/c T cell epitope and nonepitope regions in days 3, 5, and 7 p.i. in PBS, S-QIIV, and Wyeth/IL-15/5Flu in (**A**) HA, (**B**) NA, and (**C**) NP genes. Comparison between S-QIIV and Wyeth/IL-15/5Flu versus PBS within the same time point. Diversity measured in CD4^+^ and CD8^+^ T cell–depleted samples across (**D**) HA, (**E**) NA, and (**F**) NP. Comparison between nondepleted PBS and other conditions. Indicated statistical significance comparison between PBS and other conditions within the same time point (A to C) and depletion status (D to F) was determined by a Kruskal-Wallis test. False discovery rate (0.1) was determined by the original Benjamini and Hochberg method. Black annotation denotes significance between different vaccination groups at the same time point; red annotation denotes significance between different depletion status within the same vaccination group. *P ≤ 0.05, **P ≤ 0.01, and ***P ≤ 0.001.

To determine whether a T cell–activating vaccine led to targeted immune pressure against T cell epitopes, we examined diversity within known BALB/c T cell epitope (*30*) and nonepitope regions, as a negative control, were examined (Fig. 5). Nonepitope regions were selected as 40–amino acid (aa) segments, which did not overlap with known BALB/c epitope regions of 20 aa in length and within 10 aa flanking either side. Regions were assessed by analyzing the nucleotide base diversity of all aligned NGS reads within the regions (*31*). Heatmaps of diversity within known HA, NA, and NP T cell epitopes indicate that neither vaccine induces widespread enhanced mutational rates within T cell epitope regions (Fig. 5, A to C). When diversity analysis was performed on T cell–depleted groups, CD4^+^ depletion led to decreased diversity in PBS-vaccinated mice, while Wyeth/IL-15/5Flu vaccination significantly enhanced diversity by comparison across all epitope regions assessed (Fig. 5, D to F).

Wyeth/IL-15/5Flu vaccination induced an increased number of mutable positions and overall mutational frequency than S-QIIV in immunodominant epitope regions NP_55–69_, NP_147–155_, HA_140–154_, and HA_518–526_.

Mapping of mutations arising in subdominant and immunodominant BALB/c epitope regions at days 3, 5, and 7 in PBS-, S-QIIV–, and Wyeth/IL-15/5Flu–vaccinated mice showed an increase in mutations over time, primarily in Wyeth/IL-15/5Flu–vaccinated conditions (Table 2). Mutable positions at anchor positions (*32*, *33*) were more likely to arise from day 5 onward across all epitopes alongside mutations across TCR contact sites. Mutations within anchor residues can affect peptide loading into the MHC-peptide complex, but further functional analysis is needed. To assess background genetic flexibility of known BALB/c epitope regions in the absence of epitope-directed immune pressure, comparison was made between our NGS dataset and surveillance data of HA, NA, and NP sequences across multiple years, geographical locations, and host species (Table 2). Infection of mice led to common epitope variants between S-QIIV and Wyeth/IL-15/5Flu vaccination conditions. There was no similarity in dominant mutants found in mice and the reference National Center for Biotechnology Information (NCBI) database, suggesting that the most biologically fit adaptations were not arising. Furthermore, the database mutation rates were higher than those observed in mice in every epitope region, e.g., 42.7% of database sequences for HA_177–199_ versus on 0.1 to 2.2% in mice; therefore, the background genetic flexibility of epitope regions is higher than that found in our mouse dataset.

**Table 2.** Comparison of mutations arising within epitope regions in NGS datasets and NCBI consensus sequences. Mutations within HA, NA, and NP BALB/c epitopes are compared across datasets from PBS-vaccinated [d3 (*n* = 5), d5 (*n* = 5), and d7 (*n* = 9)], SQIIV-vaccinated [d7 (*n* = 4)], and Wyeth/IL-15/5Flu–vaccinated [d7 (*n* = 6)] viral lung isolates after H1N1 challenge. These sequences are compared to mutations within the same epitope regions from an NCBI dataset of randomly selected sequences (*n* = 1000). Mutable positions are highlighted in red, and overall dominant mutation within dataset is noted as WT amino acid–epitope position-mutated amino acid. * in NA_79–93_ indicates an “amber” stop mutation. N/A, not applicable.

**Protein/epitope/** **T cell restriction** **and dominance**			**Positions mutable**				**Overall mutational frequency in dataset**				**Dominant mutant**			
	**Source**	**Epitope**	**NCBI**	**PBS**	**S-QIIV**	**Wyeth/** **IL-15/5Flu**	**NCBI**	**PBS**	**S-QIIV**	**Wyeth/** **IL-15/5Flu**	**NCBI**	**PBS**	**S-QIIV**	**Wyeth/** **IL-15/5Flu**
**HA**														
HA_126–137_	d3 p.i. H1Ca04	SFERFEIFPKT		2	1	1		1	0.2	0.1		F8V	F8V	F8V
CD4^+^ BALB/c	d5 p.i. H1Ca04	SFERFEIFPKT		2	1	1		0.8	0.5	0.3		F8V	S1P	F8V
Subdominant	d7 p.i. H1Ca04	SFERFEIFPKT		1	1	3		0.2	0.3	4.6		F8V	F8V	E6G
								
	NCBI database	SFERFEIFPKT	7				2.9				T11E			
HA_140–154_	d3 p.i. H1Ca04	AKSFYKNLIWLVKKG		0	0	0		0	0	0		N/A	N/A	N/A
CD4^+^ BALB/c	d5 p.i. H1Ca04	AKSFYKNLIWLVKKG		2	2	0		1	1.3	0		I9M	S3G	N/A
Immunodominant	d7 p.i. H1Ca04	AKSFYKNLIWLVKKG		0	1	4		0	0.1	5.5		N/A	L11R	N7D
														
	NCBI database	AKSFYKNLIWLVKKG	11				29.6				S3G			
HA_177–199_	d3 p.i. H1Ca04	KLSKSYINDKGK		1	0	0		0.1	0	0		D9E	N/A	N/A
CD4^+^ BALB/c	d5 p.i. H1Ca04	KLSKSYINDKGK		4	1	3		1.1	0.1	0.6		S5F	K4T	D9G
	d7 p.i. H1Ca04	KLSKSYINDKGK		0	3	1		0	0.7	2.2		N/A	L2P	L2P
Subdominant	NCBI database	KLSKSYINDKGK	11				42.7				K1N/I7A/D9N/G11E			
HA_518–526_	d3 p.i. H1Ca04	IYSTVASSL		0	0	0		0	0	0		N/A	N/A	N/A
CD8^+^ BALB/c	d5 p.i. H1Ca04	IYSTVASSL		0	0	0		0	0	0		N/A	N/A	N/A
Immunodominant	d7 p.i. H1Ca04	IYSTVASSL		0	0	2		0	0	1.6		N/A	N/A	T4A
														
	NCBI database	IYSTVASSL	4				4.1				V5A			
**NA**														
NA_79–93_	d3 p.i. H1Ca04	VSGWAIYSKDNSVRI		1	0	1		1.9	0	0.1		G3R	N/A	I15L
CD4^+^ BALB/c	d5 p.i. H1Ca04	VSGWAIYSKDNSVRI		2	0	1		1.3	0	6.4		G3R	N/A	W4*
Subdominant	d7 p.i. H1Ca04	VSGWAIYSKDNSVRI		2	2	3		4.1	0.6	4.9		S8N	W4R	I15L
														
	NCBI database	VSGWAIYSKDNSVRI	11				51.9				I13V			
NA_191–201_	d3 p.i. H1Ca04	LKYNGIITDTI		0	0	0		0	0	0		N/A	N/A	N/A
CD4^+^ BALB/c	d5 p.i. H1Ca04	LKYNGIITDTI		0	1	2		0	0.5	1.6		N/A	D9G	N4D
Subdominant	d7 p.i. H1Ca04	LKYNGIITDTI		1	1	1		4.2	0.8	0.7		I7V	I11T	I11T
														
	NCBI database	LKYNGIITDTI	9					15.6				D9E		
**NP**														
NP_39–47_	d3 p.i. H1Ca04	FYIQMCTEL		0	0	0		0	0	0		N/A	N/A	N/A
CD8^+^ BALB/c	d5 p.i. H1Ca04	FYIQMCTEL		0	0	0		0	0	0		N/A	N/A	N/A
Subdominant	d7 p.i. H1Ca04	FYIQMCTEL		0	0	0		0	0	0		N/A	N/A	N/A
														
	NCBI database	FYIQMCTEL	6				2.1				I3V			
NP_55–69_	d3 p.i. H1Ca04	RLIQNSITIERMVLS		0	0	0		0	0	0		N/A	N/A	N/A
CD4^+^ BALB/c	d5 p.i. H1Ca04	RLIQNSITIERMVLS		1	0	1		0.2	0	0.5		I9L	N/A	V13A
Immunodominant	d7 p.i. H1Ca04	RLIQNSITIERMVLS		1	2	2		2.6	1	3.2		R1L	V13A	E10G
														
	NCBI database	RLIQNSITIERMVLS	8				15				I7L			
NP_147–155_	d3 p.i. H1Ca04	TYQRTRALV		0	0	0		0	0	0		N/A	N/A	N/A
CD8^+^ BALB/c	d5 p.i. H1Ca04	TYQRTRALV		0	0	1		0	0	0.1		N/A	N/A	V9G
Immunodominant	d7 p.i. H1Ca04	TYQRTRALV		0	0	2		0	0	2.3		N/A	N/A	T1I
														
	NCBI database	TYQRTRALV	4				0.7				Q3H			
NP_218–226_	d3 p.i. H1Ca04	AYERMCNIL		0	0	0		0	0	0		N/A	N/A	N/A
CD8^+^ BALB/c	d5 p.i. H1Ca04	AYERMCNIL		0	0	0		0	0	0		N/A	N/A	N/A
Subdominant	d7 p.i. H1Ca04	AYERMCNIL		0	2	2		0	1.1	2.9		N/A	Y2C	R4G
														
	NCBI database	AYERMCNIL	7				1.7				M5I			

**Fig. 6.**
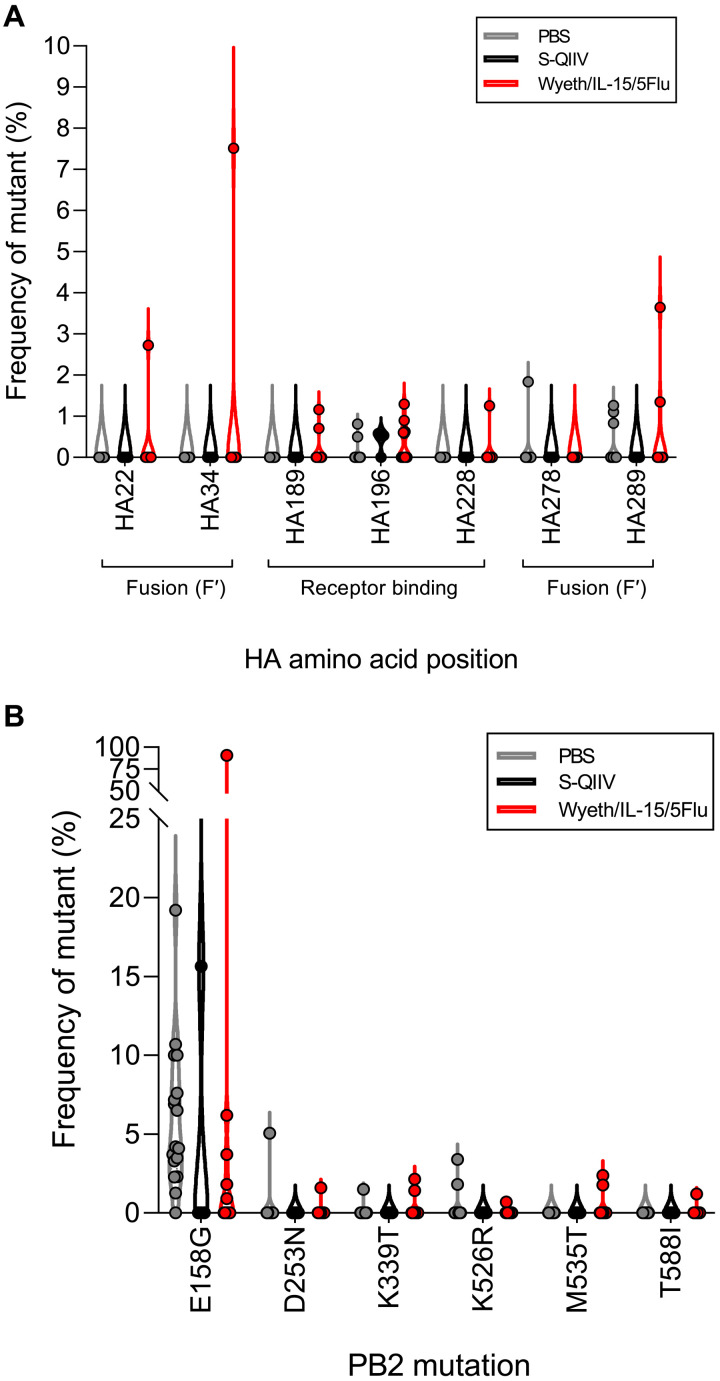
Mutations within conserved HA stem regions and beneficial PB2 adaptations can arise in Wyeth/IL-15/5Flu–vaccinated conditions. Frequency of mutant (%) arising within H1N1-challenged PBS (*n* = 17)–, S-QIIV (*n* = 4)–, and Wyeth/IL-15/5Flu (*n* = 12)–vaccinated BALB/c mice at day 7 p.i. across two separate sequencing rounds within (**A**) at functional sites within the HA stem and (**B**) known mammalian-associated pathology mutations arising in PB2. Individual data points shown on violin plot.

Analysis of mutations within functional sites of conserved HA stem regions (*34*) across two rounds of sequencing (additional sequencing read coverage can be seen in fig. S5) demonstrated that Wyeth/IL-15/5Flu–vaccinated conditions had the capacity to generate high AF breakthrough mutations within HA functional sites (Fig. 6A). The highest AF mutants arose within individual Wyeth/IL-15/5Flu–vaccinated mice at HA_22_ (2.7%, arising in 1 of 12 mice), HA_34_ (7.5%, arising in 1 of 12 mice), and HA_289_ (3.6%, arising in 2 of 12 mice) in the HA_1_ fusion domains.

Furthermore, known PB2 mutations (*35*–*37*), which enhance viral polymerase activity, shows that adapted PB2 mutants frequently arose in PBS-vaccinated mice (in 17 of 17 mice) but were also able to arise within vaccinated conditions (S-QIIV in 1 of 4 mice and Wyeth/IL-15/5Flu in 9 of 12 mice) (Fig. 6B). The PB2-E158G mutant was the most prevalently observed (in 22 of 33 mice total) and arose at the highest frequency in individual mice (range, 0.9 to 90.8%; mean, 6.7%; SD, ±15.8%), with the highest observed mutant, at 90.8%, arising in a single Wyeth/IL-15/5Flu–vaccinated mouse. The virus used for infection was derived from the H1N1 pandemic virus, was generated by reverse genetics and not serially passaged in tissue culture or mice prior, and thus may represent a mammalian adaptation of the swine-origin PB2 in mice (*38*).

Selected mutants identified from two rounds of NGS were unable to be rescued by reverse genetics after three to four serial passages per attempt (table S1). Previously identified mutants determined to be beneficial or amino acid changes (*35*–*37*, *39*) directly inside or within extra epitopic amino acid regions of BALB/c T cell epitopes were selected for generation on a phW2K plasmid backbone with A/California/04/2009 gene inserts. Round 1 mutants were unable to be rescued over four attempts, while wild-type (WT) virus was successfully rescued to an HA titer of 1:4. Round 2 mutants were unable to be rescued over five attempts, while WT virus was successfully rescued to an HA titer of 1:8. This could indicate that while the influenza virus is in the process of expanding its sequence landscape for acquiring possible adaptive mutations while under selection pressure, this may not translate into viable viruses that can be subsequently transmitted, and other factors such as comutations may be needed to sustain viability.

## DISCUSSION

Influenza viruses are adept at adaptation to selection pressure, and next-generation vaccines may have nonsterilizing function leading to inadvertent selection bottlenecks on the influenza virus genome. This study provides a new method of direct ex vivo full genome sequencing in an experimental animal model, with sufficient read coverage as late as day 7 p.i. in IAV-vaccinated conditions, which is especially relevant for comparison of standard-of-care inactivated vaccines and next-generation T cell–activating vaccines. Our observation that S-QIIV does not enhance mutations within the IAV genome mirrors the results seen previously in a study determining the effects of IIV and LAIV vaccination in healthy adults on the H3N2 genome (*40*). While it is positive that T cell–activating vaccination does not induce T cell escape variants within our dataset, there was increased variation across the influenza genome and, in some cases, high AF mutations known to have adaptive host functions, such as PB2-E158G.

Observed increases in both incidence and frequency of mutations in Wyeth/IL-15/5Flu–vaccinated conditions indicate that enhanced immune pressure driven by a T cell–activating vaccine induces more mutations on the IAV genome than traditional IIV-mediated protection. T cell depletion experiments showed that selection pressure was being driven by T cell responses in Wyeth/IL-15/5Flu–vaccinated conditions, which may indicate that T cell selection pressure could be a factor in next-generation T cell–activating vaccines, an issue that requires further investigation. Mutations arising as a result of enhanced T cell pressure appear to be stochastic in nature, with known BALB/c T cell epitope regions not displaying increased genetic diversity compared to nonepitope regions. However, it was also seen that although mutations were stochastic in nature, regions within HA stem and beneficial PB2 mammalian pathogenic mutations could still arise to potentially very high AF in individual mice (>90%). Fitness selection bottlenecks in vaccine scenarios may be tighter than unvaccinated/naïve conditions, leading to a higher threshold for virus immune escape and may propel adaptations within some individuals. Further work is needed to characterize the subsequent mutations identified.

The effect of T cell pressure on viral diversification, from our investigation, does not specifically target surface glycoproteins over more conserved internal proteins, as changes were observed throughout the genome and in proteins not encoded by the vaccine. However, increased SNP incidence and frequency arising in genes PA and PB2 may have been enhanced by lower read coverage across these genes at day 7 p.i. in Wyeth/IL-15/5Flu–vaccinated mice. While this is due to inherent biological limitations of low viral load by day 7 after nonlethal viral challenge in a vaccinated individual, appropriate quality control measures and an enhanced Phred quality score of Q33 (base call accuracy, >99.9%) were used to account for these effects. Conversely, these results are likely to be conservative estimates of the mutation load per sample, as SNPs below quality control cutoffs were discarded.

Enhanced T cell pressure did not induce mutations within NS or M gene regions, indicating that they may be unaffected by next-generation vaccines. The Wyeth/IL-15/5Flu vaccine contains both M1 and M2, and yet, there was limited sequence variation in these genes. The lack of induced mutations within the M gene further solidifies its attractiveness as an immunological target for universal vaccines and is already included in candidates undergoing clinical trials such as the MVA-NP+M1 vaccine (*5*, *41*).

Conversely, internal genes PA and PB2 are not included in the Wyeth/IL-15/5Flu vaccine yet demonstrated higher mutational incidence and frequency at day 7 p.i., potentially indicating a compensatory mechanism used by IAV to circumvent directed T cell pressure. Compensatory mechanisms in response to T cell pressure have been observed previously in other viruses such as HIV, where a dominant T cell escape mutation developed comutations to pass fitness limitations (*42*). Similar effects have been observed in IAV as a response to antiviral resistance (*43*) alongside compensatory mutations across separate genes (*44*, *45*). Epistatic interactions (*46*) across the IAV genome can also pave the way for successful variants to arise and drive evolution (*47*–*50*). However, further investigation is required to fully characterize the nature of increased mutagenesis within nontargeted genes in the context of T cell–mediated vaccination.

While estimations for human transmission bottlenecks vary, previous studies have indicated that there are low levels of intrahost diversity within the population (*40*), meaning that transmission bottlenecks may not be sufficiently large enough to transmit low-frequency variants. Current models suggest that transmission bottlenecks are tight (one to two genomes), and evolution of IAV at the host level is dominated by stochastic processes (*51*), not positive selection, and that stochastic evolution may even outcompete beneficial mutations (*52*). This could indicate that while mutational frequency may be elevated in an individual inoculated with a next-generation T cell–activating vaccine, there would be limited opportunity for interhost transmission. Experimental models have also demonstrated that while increased mutations arise later during IAV infection in healthy individuals, mutations must arise early to replicate to sufficient titers to be transmitted (*53*). However, this model also proposed that coinfection with mutant and WT viruses allowed mutant virus to predominate the upper respiratory tract, irrespective of immune status, signaling that breakthrough infections still pose a significant risk. T cell vaccines that are currently in development have also been shown to reduce viral load in experimental challenge models (*6*), which could tighten transmission bottlenecks further (*54*). However, as stochastic events can result in beneficial mutants that have increased viral replication (*55*), there is an inherent risk of potential novel variants emerging, which could either enhance pathogenesis or circumvent immunological memory.

Next-generation universal IAV vaccines can hopefully offer lasting immunity against seasonal and emerging strains with pandemic potential. Use of T cell–activating vaccines will potentially arm us against pandemic threats and significantly affect seasonal influenza mortality rates. As a universal vaccine candidate will hopefully be approved for clinical use in the near future, it is necessary to further investigate potential hurdles before they arise. Additional studies are needed to assess whether current vaccine candidates can elicit similar immune pressure in humans and whether immune pressure results in transmissible variants. Our findings indicate that heterosubtypic universal IAV vaccination imposes greater selection bottlenecks on influenza viruses than current inactivated vaccines. Full influenza virus genome-wide surveillance in the population of circulating viruses will be essential to monitor and track any successful breakthrough mutants that may become established, which could pose a risk for unvaccinated or immunocompromised individuals.

## MATERIALS AND METHODS

### Study design

BALB/c mice were twice subcutaneously vaccinated with PBS, S-QIIV, or Wyeth/IL-15/5Flu 21 days apart. On day 42 after initial vaccination, mice were challenged intranasally with H1N1. On the selected day, postinfection mice were culled, and lungs were harvested for viral RNA extraction. IAV RNA was isolated using full-genome RT-PCR using universal primers and spiked-in NA primers to generate PCR products of all eight genes. Samples of sufficient quality were submitted for next-generation Illumina NovaSeq sequencing (Fig. 1A).

### Vaccines

The T cell–activating vaccine, Wyeth/IL-15/5Flu, was previously described in detail by Poon *et al.* (*29*). Briefly, the replication-competent vaccinia Wyeth strain encodes the NP, HA, and NA proteins derived from H5N1 A/Vietnam/1203/2004 and the M1 and M2 proteins derived from A/CK/Indonesia/PA/2003. The vaccine encodes human IL-15 cytokine as a molecular adjuvant. Mice were vaccinated subcutaneously with 10^7^ plaque-forming units (PFU) in 100 μl of PBS. We also used the standard quadrivalent IIV (Sanofi Pasteur, 2016/2017 Northern Hemisphere season) containing A/California/7/2009 (H1N1)pdm09–like virus, A/Hong Kong/4801/2014 (H3N2)–like virus, B/Phuket/3073/2013, and B/Brisbane/60/2008–like virus. Mice were given a third of a human dose, equivalent to 5 μg per HA in 160 μl, which was given intramuscularly. Female BALB/c (H-2^d^) (6 to 8 weeks old) mice were vaccinated with either Wyeth/IL-15/5Flu, S-QIIV, or PBS, twice 21 days apart.

Mice were treated for in vivo depletion of CD4^+^ T cells [by monoclonal antibody (mAb) GK1.5] or CD8^+^ T cells (by mAb 2.43; both BioXCell, USA) by intraperitoneal injection at 100 μg/200 μl in PBS at days −4, −2, 0, +3, and +5 of influenza challenge (Fig. 3A). Depletion efficiency in the blood at the time of sampling was 74 to 99% compared to nondepleted mice. All experimental procedures were conducted in accordance with the standards and approved by the Committee on the Use of Live Animals in Teaching and Research, The University of Hong Kong.

### Viruses

A/California/04/2009 (H1N1) was rescued by reverse genetics, plaque purified from MDCK cells, and amplified in 10- to 12-day-old embryonated, pathogen-free chicken eggs. Mice were challenged intranasally with 0.1 LD_50_ equivalent (2.14 × 10^3^ TCID_50_/25 μl of H1N1).

### Full-genome IAV NGS from infected mouse lungs

Viral RNA extraction was performed on mouse lung homogenate to isolate IAV RNA for NGS. Lungs were washed in PBS and homogenized (Omni International, USA) on ice, and supernatant was clarified by centrifugation. The viral load of lung homogenates from individual mice was determined using standard TCID_50_ assay on MDCK cells, as previously described (*30*).

Lung supernatant homogenate (140 μl) was added to 560 μl of AVL viral lysis buffer with added RNA carrier (Qiagen, Germany) to inactivate viral particles and preserve RNA. The lung homogenate AVL sample was thoroughly vortexed and incubated at room temperature (RT) for 10 min. For isolation and purification of RNA, a QIAamp Viral Mini Kit (Qiagen) was used according to the manufacturer’s “spin protocol” and eluted in 60 μl of AVE viral RNA elution buffer and stored at −80°C.

RT-PCR for the amplification of the eight IAV genes from viral RNA samples used qScript XLT One-Step RT-PCR Kit (QuantaBio, USA), with a proofreading polymerase enzyme. For RT-PCR reaction, 2 μl of viral RNA sample was added to 25 μl of 2x reaction buffer, Uni12 Inf-1 (80 nM; GGGGGGAGCAAAAGCAGG), Uni12 Inf-3 (120 nM; GGGGGGAGCGAAAGCAGG), Uni13 Inf-1 (200 nM; CGGGTTATTAGTAGAAACAAGG), NA-1 F (120 nM; TATTGGTCTCAGGGAGCAAAAGCAGGAGT), NA-1413R (120 nM; ATATGGTCTCGTATTAGTAGAAACAAGGAGTTTTTT), and MilliQ water and amplified by PCR [RT: 48°C 20 min, 94°C 3 min; PCR1: 94°C 30 s, 45°C 30 s, 68°C 3 min (×5 cycles); PCR2: 94°C 30 s, 58°C 30 s, 68°C 3 min (×31 cycles)]. Universal primers were used at varying concentrations as based on the protocol developed by McGinnis *et al.* (*56*).

Products of the RT-PCR were visualized on a 1.5% TAE–agarose gel, prestained with SYBR safe (1:1000; Thermo Fisher Scientific), alongside Invitrogen 1 Kb Plus DNA Ladder (Thermo Fisher Scientific). Gels were run at 100 V for 60 min, and the image was then captured using a Gel Doc EZ System (Bio-Rad, USA). The DNA concentration of PCR products from gels was determined for quality control (Fig. 1F). RT-PCR products were purified by QIAamp Viral Mini Kit (Qiagen, Germany) according to the manufacturer’s instructions. Samples were sequenced by Illumina NovaSeq (PE150) at an average sequence depth of >1000 base pairs. Sample library was prepared by Centre for PanorOmic Sciences (CPOS), University of Hong Kong using the Nextera XT kit (Illumina, USA).

Our analysis pipeline used Trimmomatic for quality control read trimming and removal of low-quality reads (version 0.36 with parameters “PE -threads 48 -phred33 SLIDINGWINDOW:4:20 MINLEN:40”). Sequences were then aligned to reference genome using BWA-MEM (version 0.7.5a-r405 with default parameters). The AFs (proportion of gene copies of an allele in a defined population) at all nucleotide positions in reference to the influenza genome (A/California/04/2009, GISAID ID: EPI_ISL_29573) were counted from the bam file from BWA-MEM by Samtools mpileup (v1.11) and mpileup2readcounts (https://github.com/gatoravi/mpileup2readcounts). To filter errors, variant calling was performed using a combination of sensitive variant callers (with minimum AF of 0.005 if available): LoFreq (v2.1.2), FreeBayes (v1.3.1, 0.005), and VardictJava (v1.6) (*57*). The detailed commands and parameters for executing LoFreq, FreeBayes, and Vardict can be found via https://github.com/Koohoko/T-cell-activating-vaccines-flu-mutation/blob/main/scripts/NGS_resequencing_PE.sh. For background genetic flexibility assessment, NCBI surveillance sequences (*n* = 1000) were randomly selected from the NCBI Influenza Virus Database (NCBI, USA) from H1N1 sequences across multiple hosts, regions, and collection dates. Sequences were subsequently aligned using MAFFT (v7.455 with default parameters). In addition to incidence of SNPs, cumulative AF was used to determine the mean within-host mutational load by summing the AFs of all identified SNPs in a sample, as applied similarly in other studies (*58*, *59*).

### Assessing adaptive immune responses from vaccination

To assess influenza-specific T cell responses from vaccination, the CD4^+^ T cell and CD8^+^ T cell response of BAL was determined by IFN-γ ICS assay, stimulated with 4 Multiplicity of Infection (MOI) of ultraviolet-irradiated H1N1Ca04, as previously described (*30*).

To assess influenza-specific binding antibodies from vaccination, IgG responses specific for H1N1 representative HA and NP recombinant proteins (Sino Biological, China) were determined by enzyme-linked immunosorbent assay (ELISA), as previously described (*60*). Data from flow cytometry and ELISA IgG responses with background subtracted are indicated.

### Reverse genetics

Reverse genetics was used to generate H1N1 variants identified by NGS to determine the phenotype of de novo mutants. PhW2k (ampicillin-resistant) plasmids encoding the full eight gene segments of A/California/04/2009 were used to generate WT viruses. Selected mutant plasmids were generated (Genewiz, USA) and substituted WT plasmid where appropriate. Cocultured 293T and MDCK cells were transfected (500 ng per plasmid) using TransIT-LT1 transfection reagent (Mirus Bio, USA). HAI (haemagglutin inhibition) assays were performed to assess successful virus rescue. Supernatant was used to inoculate T75 flasks of MDCK and specific pathogen–free eggs for three to four rounds of passage.

### Statistical analysis

Data were visualized using GraphPad Prism software. Statistical significance of mouse weight loss was determined by repeated-measures two-way analysis of variance (ANOVA) with Geisser-Greenhouse correction and Šidák’s multiple comparisons test. All other statistical analyses were performed using an unpaired nonparametric one-way ANOVA Kruskal-Wallis test and adjusted for multiple comparisons. The false discovery rate was calculated by the original Benjamini and Holchberg method and set at a false discovery rate of 0.1. For sequencing data, ≥0.01 AF was used as a background cutoff, and comparisons between vaccination groups, time points, and immune conditions were performed and adjusted for multiple comparisons on a gene-by-gene basis.
