# Supplementary material for: Next-generation T cell–activating vaccination increases influenza virus mutation prevalence
Source: Sci Adv. 2022 Apr 6;8(14):eabl5209. doi: 10.1126/sciadv.abl5209 (PMC8986104; doi:10.1126/sciadv.abl5209)
Supplement: Supplementary file 1 — Figs. S1 to S5 Table S1 [file sciadv.abl5209_sm.pdf]

Science Advances
AAAS

Supplementary Materials for

Next-generation T cell–activating vaccination increases influenza virus mutation prevalence

Maireid B. Bull, Haogao Gu, Fionn N. L. Ma, Liyanage P. Perera, Leo L. M. Poon, Sophie A. Valkenburg*

*Corresponding author. Email: sophie.v@unimelb.edu.au

Published 6 April 2022, *Sci. Adv.* **8**, eabl5209 (2022)
DOI: 10.1126/sciadv.abl5209

This PDF file includes:

Figs. S1 to S5
Table S1

Supplementary Figures:

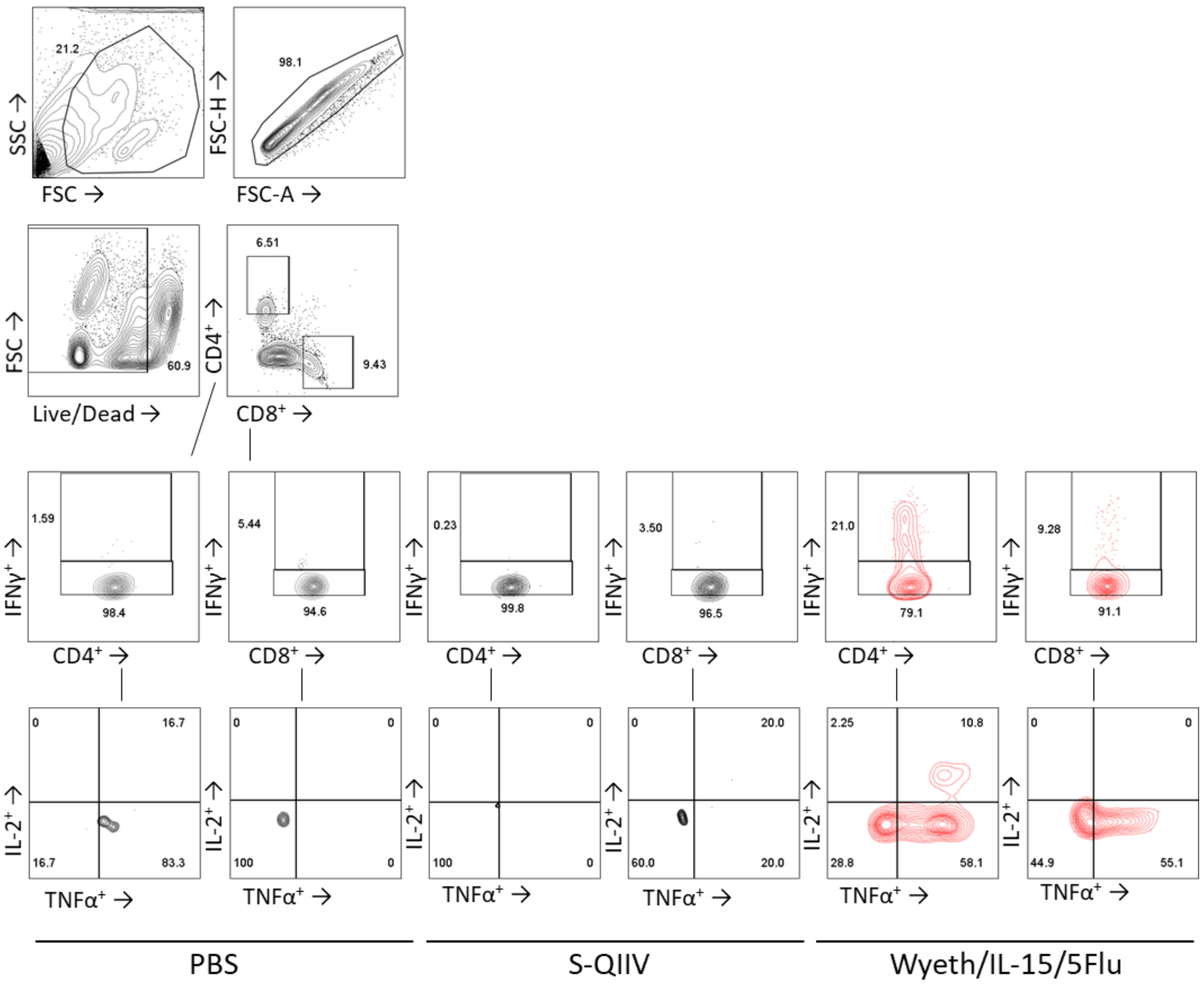

Figure S1: Flow Cytometry gating strategy to characterise T cells isolated from BAL Representative FACS plots showing the gating strategy of identifying IFNγ^{+} IL-2^{+} and TNFα^{+} producing CD4^{+} and CD8^{+} T cell populations in PBS (grey), S-QIIV (black) and Wyeth/IL-15/5Flu (red) vaccinated mice at day 7 H1N1 challenge.

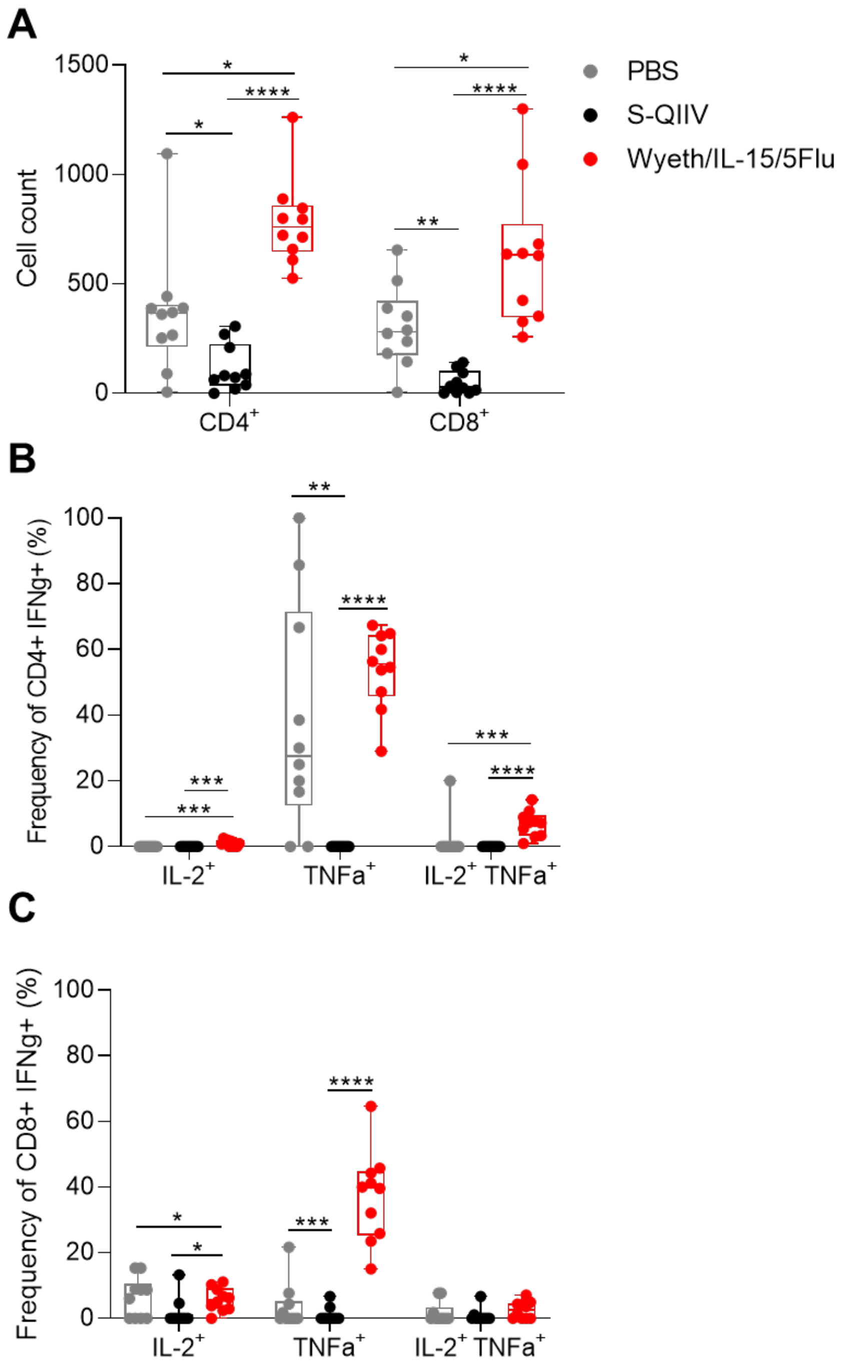

Figure S2: Cytokine production of vaccine induced T cell responses during infection ICS of T cell populations isolated from BAL fluid from PBS, S-QIIV and Wyeth/IL-15/5Flu at day 7 post H1N1 challenge (n=10). Showing **(A)** $CD4^+$ and $CD8^+$ T cell counts, **(B)** multicytokine producing $CD4^+$ T cells, and **(C)** multicytokine producing $CD8^+$ T cells. Individual data points shown on box and whiskers plot, showing mean and upper and lower quartiles with min-max range. Statistical significance was determined by a Kruskal-Wallis test. False discovery rate (0.1) was determined by the original Benjamini and Hochberg method. * P <0.05, ** P <0.01, *** P <0.005, **** P <0.0001.

A

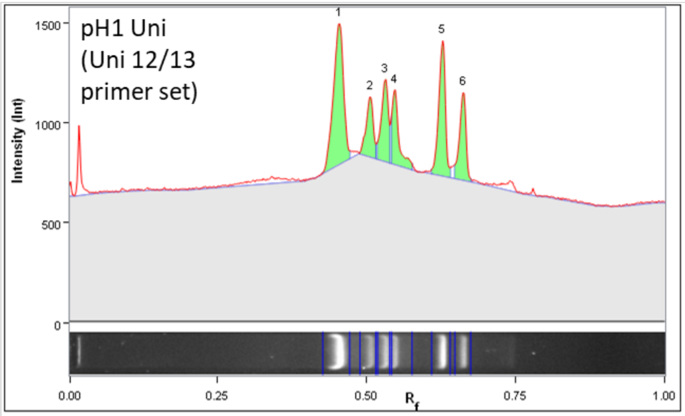

Band No.	Band Label	Base Pairs (bp)	Relative Front	Adj. Volume (Int)	Volume (Int)	Abs. Quant. (ng)	Rel. Quant.	Band %	Lane %
1		1,679.5	0.455	590,733	2,117,979	56.2	N/A	32.2	26.3
2		1,326.8	0.506	182,529	1,210,230	23.7	N/A	9.9	8.1
3		1,213.5	0.531	237,864	973,488	28.1	N/A	13.0	10.6
4		1,148.1	0.547	213,588	1,429,836	26.2	N/A	11.6	9.5
5		906.6	0.628	373,065	1,376,439	38.9	N/A	20.3	16.6
6		770.9	0.663	236,793	1,070,235	28.0	N/A	12.9	10.5

B

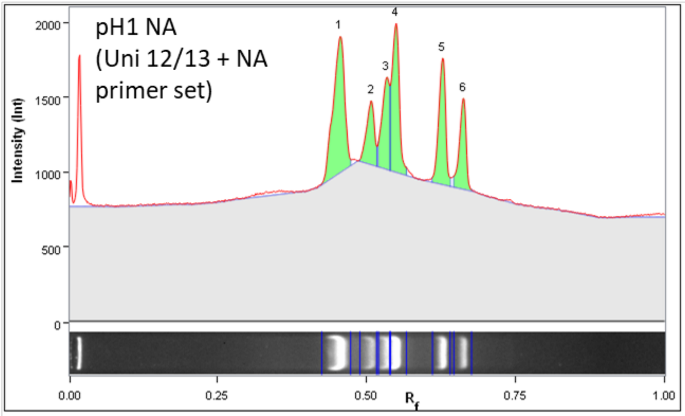

Band No.	Band Label	Base Pairs (bp)	Relative Front	Adj. Volume (Int)	Volume (Int)	Abs. Quant. (ng)	Rel. Quant.	Band %	Lane %
1		1,662.3	0.456	712,266	2,412,606	65.9	N/A	29.1	24.2
2		1,319.4	0.508	232,458	1,321,614	27.7	N/A	9.5	7.9
3		1,200.0	0.534	316,608	1,039,074	34.4	N/A	12.9	10.8
4		1,135.5	0.550	504,798	1,529,031	49.4	N/A	20.6	17.2
5		906.6	0.628	403,869	1,400,919	41.3	N/A	16.5	13.7
6		770.9	0.663	280,092	1,241,952	31.5	N/A	11.4	9.5

Figure S3: Spiking of NA specific primers improves RT-PCR NA product quantity Comparison between gene segment estimated quantity determined using BioRad Image Lab between **(A)** Uni12/13 primer set and **(B)** Uni12/13 + NA primer set. NA band is at band position 4.

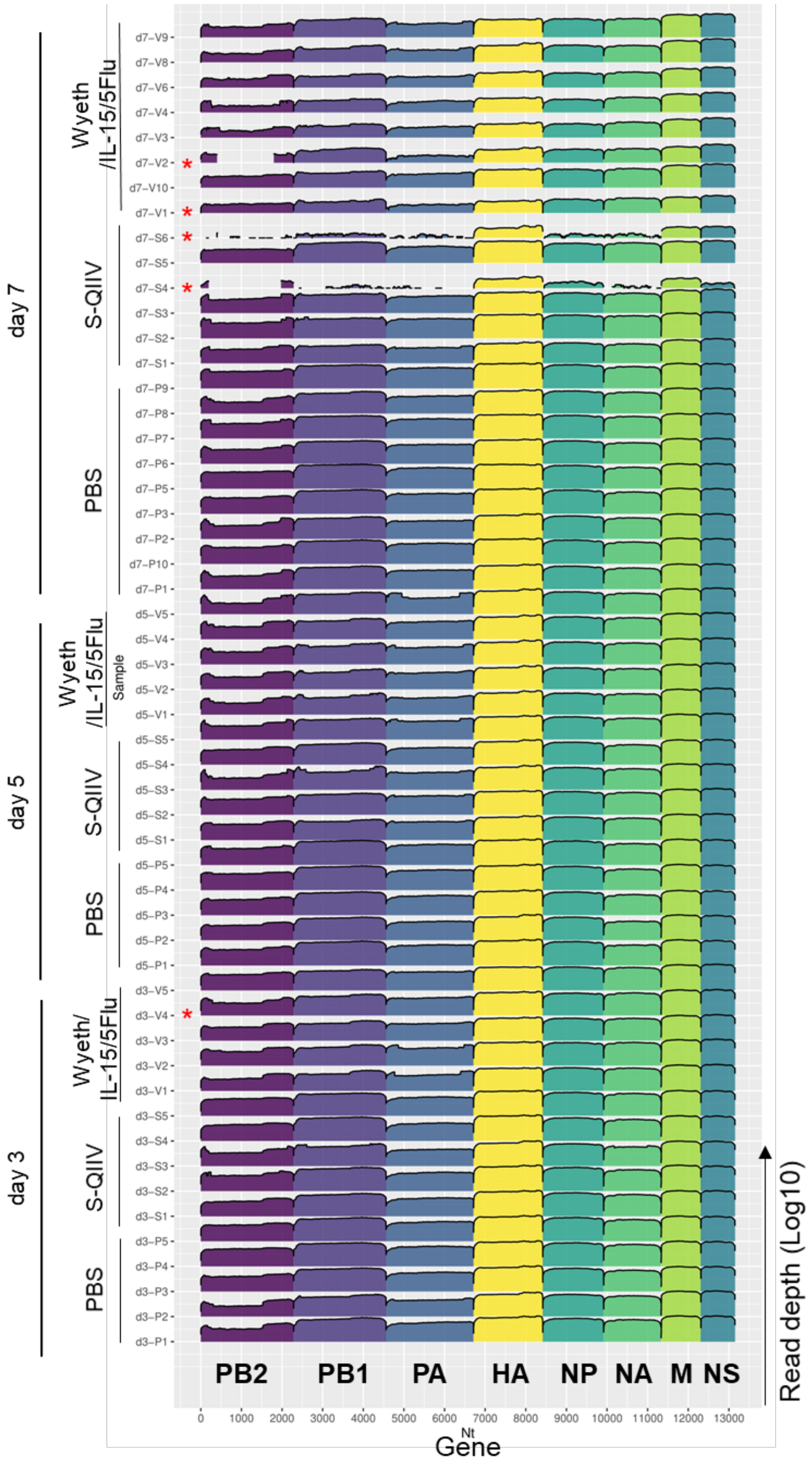

Figure S4: Read depth and coverage for 8 IAV genes for RT-PCR products Round 2 NGS read depth across the H1N1 genome from RT-PCR products by NGS from day 3, day 5 and day 7 p.i. mice. Low quality reads removed using Trimmomatic and aligned to H1N1 (Ca04/09) reference genome using BWA-MEM. Data represents individual mice and samples annotated by

‘*’ indicates a sample removed from further analysis due to either <100 read coverage or <0.8 Q33 Phred Score. Data was visualised using R.

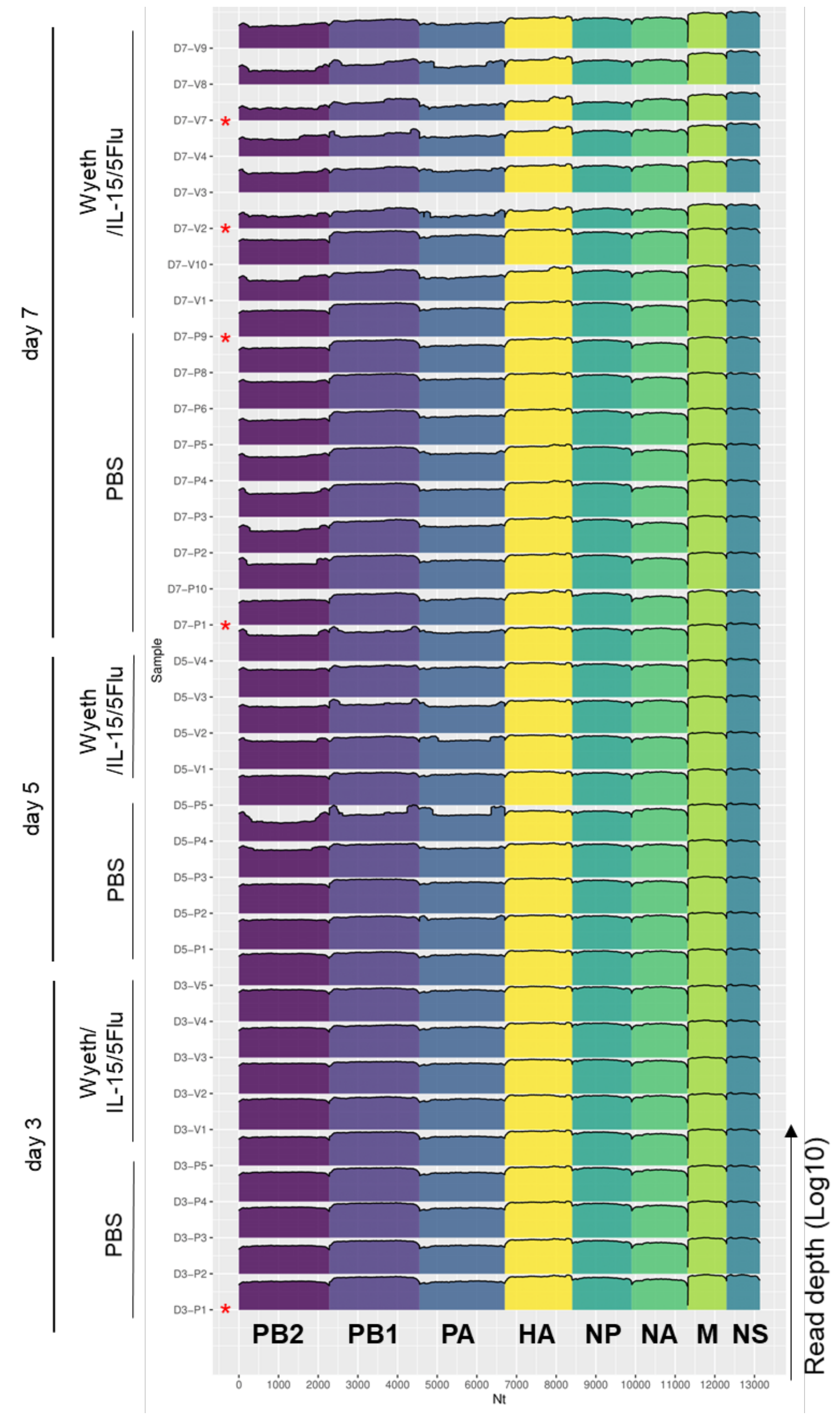

Figure S5: Read depth and coverage for 8 IAV genes for RT-PCR products Round 1 NGS read depth across the H1N1 genome from RT-PCR products by NGS from day 3, day 5 and day 7 p.i. mice. Low quality reads removed using Trimmomatic and aligned to H1N1 (Ca04/09) reference genome using BWA-MEM. Data represents individual mice and samples annotated by

'*' indicates a sample removed from further analysis due to either <100 read coverage or <0.8 Q33 Phred Score. Data was visualised using R.

Table S1: Reverse Genetics attempts of previously defined beneficial mammalian adaptations and mutations within BALB/c T cell epitope regions

Reverse Genetics						
**Round**	**Epitope and mutation**	**Location**	**T cell restriction**	**Mutational Frequency in NGS (%)**	**Vaccination group**	**HA titre**
1	WT-Ca04					1:4
	HA_{518} V5I	In Epitope	$CD8^+$	1.3	PBS	Not rescued
	NP_{39} Y2*	In Epitope	$CD8^+$	0.9	Wyeth/IL-15/5Flu	Not rescued
	NP_{55} R1Q	In Epitope	$CD4^+$	1.1	Wyeth/IL-15/5Flu	Not rescued
	NP_{147} R4K	In Epitope	$CD8^+$	1	Wyeth/IL-15/5Flu	Not rescued
	NP_{147} R6C	In Epitope	$CD8^+$	3.9	Wyeth/IL-15/5Flu	Not rescued
2	WT-Ca04					1:8
	HA_{177} K4T	In Epitope	$CD4^+$	1	PBS	Not rescued
	NP_{55} E81K	11aa outside	$CD4^+$	6.6	Wyeth/IL-15/5Flu	Not rescued
	NP_{147} D157N	2aa outside	$CD8^+$	1	Wyeth/IL-15/5Flu	Not rescued
	NP_{218} V217I	1aa outside	$CD8^+$	1.1	Wyeth/IL-15/5Flu	Not rescued
	PA T97I	Non-epitope	n/a	18.4	PBS, Wyeth/IL-15/5Flu	Not rescued
	PB2 E158G	Non-epitope	n/a	90.8	PBS, Wyeth/IL-15/5Flu	Not rescued

Note: Mutants selected for virus rescue by reverse genetics were based on 3 criteria, either: *1*) high allele frequency (>0.9%) mutants from Table 2. *2*) Mutations outside of epitope regions, but within 15aa of the epitope and above 1% AF were also selected for rescue. Or *3*) high frequency polymerase mutants in non-epitope regions were observed in our study and previous studies *(37–41)*. * indicates 'amber' stop mutation.
